# The association between skipping breakfast and cardiovascular disease: a meta analysis

**DOI:** 10.3389/fcvm.2025.1565806

**Published:** 2025-12-01

**Authors:** Hui Zhang, Shipeng Zhang, Yifan Liu, Xinyue Wang, Jinming Hu

**Affiliations:** 1 Chengdu University of Traditional Chinese Medicine, Chengdu, China; 2 Hospital of Chengdu University of Traditional Chinese Medicine, Chengdu, China

**Keywords:** skipping breakfast, cardiovascular disease, coronary artery disease, stroke, cardiovascular disease mortality, meta-analysis

## Abstract

**Background:**

Cardiovascular disease (CVD) is a global health challenge significantly influenced by healthy behaviors. Nutritional research highlights the critical role of eating habits in the development of CVD. However, existing studies on the association between skipping breakfast and CVD have produced conflicting results. To address this controversy, we conducted a meta-analysis to clarify the relationship.

**Method:**

We performed a systematic search of the PubMed, Embase, Cochrane and Web of Science databases for studies published before October 2024 to identify and assess prospective research on the relationship between skipping breakfast and CVD. In the study selection process, the PECOS framework and stringent inclusion/exclusion criteria were applied. The quality of the initially included studies was independently assessed using the Newcastle-Ottawa Scale (NOS). Data from the included studies, including odds ratio (OR) and 95% confidence intervals (CI), were extracted and analyzed using Stata 16.0. Sensitivity analyses were conducted to validate the results. Heterogeneity was assessed using the I^2^ and Cochrane Q tests, and publication bias was evaluated using Egger's test and funnel plot analysis.

**Results:**

This meta-analysis includes 2,383,813 participants. As a result, nine studies included 13 data points. Skipping breakfast, compared to regular breakfast consumption, was associated with an increased risk of CVD (OR: 1.17, 95% CI:1.09–1.26). Cardiovascular diseases were further categorized into coronary artery disease (CAD), stroke, and cardiovascular disease mortality (CDM). Skipping breakfast was associated with an increased risk of CAD (OR: 1.14, 95% CI: 1.05–1.24), stroke (RR: 1.15, 95% CI: 1.01–1.3), and CDM (OR: 1.49, 95% CI: 1.20–1.84).

**Conclusion:**

Skipping breakfast is significantly associated with increased CVD risk. Our analysis elucidates multiple pathophysiological mechanisms underlying this association. These findings collectively suggest that regular breakfast consumption may confer cardiovascular protective benefits.

**Systematic Review Registration:**

https://www.crd.york.ac.uk/PROSPERO/view/CRD42025630303, PROSPERO CRD 42025630303.

## Introduction

Breakfast is widely regarded as the most important meal of the day. However, the global trend of skipping breakfast is on the rise (1). Numerous studies suggest that a decline in the quality of the daily diet, particularly when breakfast is omitted, can negatively impact health over time (2). For instance, skipping breakfast is associated with an increased prevalence of several cardiovascular and metabolic risk factors, including overweight and central obesity, hypertension, glucose intolerance, and elevated cholesterol levels (3). Skipping breakfast is associated with decreased consumption of whole grains and reduced satiety, leading to impaired insulin sensitivity and subsequent glucose intolerance (4). Delayed breakfast timing disrupts peripheral circadian rhythms and modulates the expression of core clock genes involved in lipid metabolism (including CLOCK and BMAL1), potentially increasing the risk of dyslipidemia (5). Chronic breakfast skipping elevates pro-inflammatory mediators associated with hypertension (6–9). Skipping breakfast disrupts clock gene expression, impairs the liver's survival mechanism for supplying glucose and amino acids, elevates hunger levels, and consequently increases the risk of obesity. Furthermore, significant interrelationships exist among overweight status, hypertension, glucose intolerance, and dyslipidemia. For instance, weight gain impairs postprandial insulin secretion and blunts intestinal insulin response, resulting in compromised glycemic control. Current evidence indicates that diabetes mellitus frequently contributes to dyslipidemia development (10). Moreover, overweight and obesity constitute well-established primary risk factors for hypertension (11). As a modifiable risk factor, regular breakfast consumption may play a significant role in cardiovascular prevention (12).

There is a significant relationship between skipping breakfast and the increased risk of cardiovascular disease. However, most findings on the health effects of skipping breakfast are based on observational studies (13), which often yield inconsistent results. For example, a prospective cohort study conducted in Japan reported a significant correlation between skipping breakfast and stroke but found no significant association with coronary heart disease (CHD) (14). Similarly, a U.S.-based prospective cohort study revealed that individuals who never ate breakfast had a higher risk of both heart disease-specific and stroke-specific mortality, even after adjusting for age, gender, and race/ethnicity. In fully adjusted models, the association between skipping breakfast and heart disease-specific mortality was attenuated and became less significant, whereas the association with stroke-specific mortality remained significant (15). Additionally, a 14.9-year cohort study found an inverse association between total dietary diversity score (DDS) and CDM in females but not in males (16).

Current evidence does not conclusively support the purported benefits of breakfast omission, and the causal relationship between this practice and cardiovascular outcomes remains uncertain (17). This study examined the association between breakfast skipping and CAD, stroke, and CDM.The discussion section elucidates potential mechanisms, suggesting that breakfast omission may elevate cardiovascular risk through impaired insulin secretion and increased pro-inflammatory markers.

### Search strategy

The meta-analysis was conducted in accordance with the PRISMA statement, which examines the association between skipping breakfast and CVD. The research protocol has been registered with PROSPERO, the international registry for systematic reviews (Registration No. CRD 42025630303).

A systematic search of the PubMed, Embase, and Web of Science databases was conducted to identify Observational studies published before October 2024. The focus was on examining the relationship between skipping breakfast and the risk of CVD. PubMed search terms were: ((((((((((((((Fasting, Intermittent[Title/Abstract]) OR (Time Restricted Feeding[Title/Abstract])) OR (Feeding, Time Restricted[Title/Abstract])) OR (Time Restricted Feedings[Title/Abstract])) OR (Time Restricted Fasting[Title/Abstract])) OR (Fasting, Time Restricted[Title/Abstract])) OR (Restricted Fastings, Time[Title/Abstract])) OR (Time Restricted Eating[Title/Abstract])) OR (Eating, Time Restricted[Title/Abstract])) OR (Meal Skipping[Title/Abstract])) OR (Skipping, Meal[Title/Abstract])) OR (Breakfast Skipping[Title/Abstract])) OR (Skipping, Breakfast[Title/Abstract])) OR (“Intermittent Fasting"[Mesh])) AND ((“Cardiovascular Diseases”[Mesh]) OR ((((((((((Cardiovascular Disease[Title/Abstract]) OR (Disease, Cardiovascular[Title/Abstract])) OR (Cardiac Events[Title/Abstract])) OR (Cardiac Event[Title/Abstract])) OR (Event, Cardiac[Title/Abstract])) OR (Adverse Cardiac Event[Title/Abstract])) OR (Adverse Cardiac Events[Title/Abstract])) OR (Cardiac Event, Adverse[Title/Abstract])) OR (Cardiac Events, Adverse[Title/Abstract])) OR (Major Adverse Cardiac Events[Title/Abstract]))), Relevant reference lists were also thoroughly reviewed. The search was restricted to studies published in English.

### Eligibility criteria

According to the recommendation (18), The scope of a systematic review is determined by four key elements: population characteristics, intervention types (including comparators), outcome measures of interest, and study design. The PECOS framework (Population, Interventions, Comparators, Outcomes, and Study design) provides a structured approach to defining these parameters.The study compared individuals who did not eat breakfast (e) with those who did (C) in terms of the prevalence of CVD. The outcomes of interest were the prevalence and mortality rates for CAD, stroke, and CDM (O). The focus was solely on observational studies and research (S).

The study compared individuals who did not eat breakfast (e) with those who did (C) in terms of the prevalence of CVD. The outcomes of interest were the prevalence and mortality rates for CAD, stroke, and CDM (O). The focus was solely on observational studies and research (S).

The following criteria were included: (1) human studies; (2) observational study (cohort, case-control or cross-sectional studies); (3) examination of breakfast frequency or breakfast skipping (Skipping breakfast is defined as consuming breakfast less than five times a week or not at all.) as exposure; (4) cardiovascular events or mortality as outcomes; (5) RR, HR or OR with a 95% CI; and (6) publication in English. Studies such as comments, letters, editorials, and case reports were excluded. If the same cohort was reported in multiple publications, the study with the longest follow-up period was selected.

### Study selection

The literature screening process consisted of two stages. First, two authors (ZH and ZSP) performed a comprehensive search for relevant studies. All retrieved articles were imported into EndNote X9, and duplicates were removed using both automated and manual methods. Eligible studies were selected by screening titles and abstracts based on predefined inclusion and exclusion criteria. In the second stage, studies with uncertain eligibility underwent full-text review to assess their suitability for the meta-analysis. Disagreements were resolved through discussion with a third author (LYF) to reach a consensus.

### Data extraction and quality evaluation

The research was systematically reviewed to independently extract the following information (Table 1): author, year of publication, sample size, number of cases, gender, follow-up duration, exposure, exposure measurement, outcome, data source, study design, reporting of risk ratios (RR), hazard ratios (HR), or odds ratios (OR), along with their corresponding 95% confidence intervals (CI), and adjustment factors. For studies presenting multiple adjustment models, the RR, HR, or OR with the most comprehensive adjustment was selected. The quality of each study was evaluated using the Newcastle-Ottawa Scale (Table 2) (19), which awards a maximum of nine stars. Studies scoring more than six stars were classified as high-quality. Data extraction and quality assessment were conducted independently by two investigators, with any discrepancies resolved through discussion involving additional reviewers. In cases where multiple cardiovascular outcomes were reported within the same study, relevant outcomes were analyzed simultaneously if data on overall cardiovascular events were unavailable.

**Table 1:** Basic information of literature.

Author, year	Follow-up time(years)	Male (%)	Total sample size	The CVD cases	Data source	Measurement of exposure	Covariates	Outcome	Exposure	OR (95%CI)	Types of research
Cahill et al. (2013) (25)	1992–2008	Male (100%)	26,902	1,527	The Health Professionals Follow-up Study	Questionnaire	Energy intake (quintiles of kilocalories/day), alcohol intake (0, 0.1–<5, 5–<15, 15–<30, 30+ g/day), diet quality using the 2010 AHEI (quintiles of score), physical activity (quintiles of MET-hours/week), television watching (asked in categories 0–1.5, 2.0–6.0, 7.0–20.0, ≥21.0 h/week), sleep (<7, 7–8, >8 h/24 h), smoking status (never, past, current), marital status (married, not married), full-time work status (yes, no), had a physical exam in last two years (yes, no) and family history of CHD before the age of 60 (yes, no)	CAD	Breakfast skipping^a^	1.25 (1.03–1.51)	Cohort research
Kaneko et al. (2021) (26)	2005–2018	Male (58.7%)	2,052,108	3,771	JMDC	Questionnaire	Non-optimal eating behaviors, age, sex, BMI, waist circumference, hypertension, diabetes mellitus, dyslipidemia, and cigarette smoking	CAD	Breakfast skipping^a^	1.12 (1.02–1.24)	Cohort research
Kubota et al. (2016) (14)	1995–2010	Male (46.73%)	82, 772	66	JPHC	Questionnaire	Age, sex, body mass index, use of medication for hypertension, hypercholesterolemia, and diabetes mellitus (yes or no), history of diabetes mellitus, smoking status, regular leisure-time sports or physical exercise, sleep duration, 11–13 perceived mental stress, living alone, physical labor, and public health center areas, alcohol intake, quintiles of total energy intake, vegetables, fruits, fish, soy, milk/dairy products, nuts, saturated fatty acids, dietary fiber, and sodium.	CAD	Breakfast skipping^a^	0.95 (0.62–1.44)	Cohort research
Sharma et al. (2018) (28)	2016–2017	Male (85.69%)	1,607	980	Tertiary cardiac care hospital of Western India	Questionnaire	Basic demographic information, details of comorbidities, past medical history, individual income, smoking habit, body mass index (BMI) and physical activity levels (</≥30 min) were collected for all participants who were approached.	CAD	Breakfast skipping^a^	1.348 (1.07–1.689)	Cross-sectional research
Tada et al. (2018) (24)	2014	Male (35.4%)	47,842	-	Kanazawa Medical Association.	Questionnaire	Age, sex, hypertension, diabetes, lipid-lowering therapy, body mass index (BMI), and waist circumference	CAD	Breakfast skipping^a^	1.06 (0.91–1.24)	Cohort research
Kaneko et al. (2013) (26)	2005–2018	Male (58.7%)	2,052,108	14,223	JMDC	Questionnaire	Non-optimal eating behaviors, age, sex, BMI, waist circumference, hypertension, diabetes mellitus, dyslipidemia, and cigarette smoking	Stroke	Breakfast skipping^a^	1.1 (1.04–1.16)	Cohort research
Kubota et al. (2016) (14)	1995–2010	Male (46.73%)	82,772	101	JPHC	Questionnaire	Age, sex, body mass index, use of medication for hypertension, hypercholesterolemia, and diabetes mellitus (yes or no), history of diabetes mellitus, smoking status, regular leisure-time sports or physical exercise, sleep duration, 11–13 perceived mental stress, living alone, physical labor, and public health center areas, alcohol intake, quintiles of total energy intake, vegetables, fruits, fish, soy, milk/dairy products, nuts, saturated fatty acids, dietary fiber, and sodium.	Stroke	Breakfast skipping^a^	1.36 (1.1–1.7)	Cohort research
Rong et al. (2019) (15)	1988–1994	Male (48.0%)	6,550	8	NHANES III	Questionnaire	Age, sex, race/ethnicity, family income, smoking status, alcoholic intake, and physical activity	Stroke	Breakfast skipping^a^	3.39 (1.4–8.24)	Cohort research
Sakai et al. (2023) (29)	2008–2017	Male (76.8%)	132,795	1,165	Medical health Checkup program conducted by Panasonic Corporation	Questionnaire	Sex, age, high blood pressure, high triglyceride, low HDL-C, high fasting plasma glucose, smoking status, exercise habits, and eating behaviors at baseline	Stroke	Breakfast skipping^a^	1.12 (0.97–1.29)	Cohort research
Tada et al. (2018) (24)	2014	Male (35.4%)	47,842	6,232	Kanazawa Medical Association.	Questionnaire	Age, sex, hypertension, diabetes, lipid-lowering therapy, body mass index (BMI), and waist circumference	Stroke	Breakfast skipping^a^	0.99 (0.82–1.2)	Cross-sectional research
Rong et al. (2019) (15)	1988–1994	Male (48.0%)	6,550	41	NHANES III	Questionnaire	Age, sex, race/ethnicity, family income, smoking status, alcoholic intake, and physical activity	CDM	Breakfast skipping^a^	1.87 (1.14–3.04)	Cohort research
Sun et al. (2023) (27)	1999–2014	-	24,011	878	NHANES	Questionnaire	Age, gender, race and ethnicity, education, annual household income, smoking status, and physical activity	CDM	Breakfast skipping^a^	1.4 (1.09–1.78)	Cohort research
Xie et al. (2021) (30)	1988–1994	Male (46.6%)	9,226	603	NHANES	questionnaire	age, sex, race/ethnicity, total energy intake, overall diet quality indicated by Healthy Eating Index, percentage of carbohydrate intake at breakfast per day and percentage of dietary fibre intake at breakfast per day,serum cotinine, alcohol intake, physical activity, family income level, education, central obesity, hypertension, dyslipidaemia, T2DM status	CDM	breakfast skipping^a^	1.526 (0.701–3.326)	Cohort research

a Skipping breakfast is defined as consuming breakfast less than five times a week or not at all.

**Table 2:** Quality of the included studies.

No.	Study (year)	Score									
		Selection				Comparability	Outcome			Total	Quality
		1	2	3	4	5	6	7	8		
1	Cahill et al. (2013) (25)		★	★	★	★★	★	★	★	8	High quality
2	Kaneko et al. (2021) (26)	–	★	★	★	★★	★	★	★	8	High quality
3	Kubota et al. (2016) (14)	★	★	★	★	★★	★	★	★	9	High quality
4	Rong et al. (2019) (15)	★	★	★	★	★★	★	★	★	9	High quality
5	Sakai et al. (2023) (29)	-	★	★	★	★★	★	★	★	8	High quality
6	Sharma et al. (2018) (28)	-	★	★	–	★★	–	★	–	5	Moderate quality
7	Sun et al. (2023) (27)	★	★	★	★	★★	★	★	★	9	High quality
8	Tada et al. (2018) (24)	★	★	★	–	★★	★	★	★	8	High quality
9	Xie et al. (2022) (30)	★	★	★	★	★★	★	★	★	9	High quality

Thresholds for converting the Newcastle-Ottawa scales to AHRQ standards (high, Moderate, and low):

High quality: ★★★or ★★★★ in selection domain AND ★ or ★★in comparability domain AND★★ or ★★★in outcome/exposure domain.

Moderate quality: ★★ stars in selection domain AND ★ or ★★stars in comparability domain AND★★ or ★★★stars in outcome/exposure domain.

Low quality:—or ★ in selection domain OR—in comparability domain OR—or★ stars in outcome/exposure domain.

### Statistical analysis

In studies examining low-probability events, odds ratios (OR), hazard ratios (HR), and relative risks (RR) are often used interchangeably. This meta-analysis employed ORs and their 95% confidence intervals (CI) as the effect size to evaluate the association between skipping breakfast and cardiovascular disease (CVD), thereby accounting for variations across studies. Heterogeneity was assessed using Cochran's *Q* test (with significance set at *P* < 0.10) and quantified via the *I*^2^ statistic (20). Primary analyses were conducted using a fixed-effects model (Mantel–Haenszel method), while a random-effects model was applied in cases of significant heterogeneity (*P* < 0.10) (21). To assess the relative influence of individual studies on the pooled estimate, sensitivity analyses were performed by systematically omitting one study at a time. Publication bias was evaluated through visual inspection of funnel plot symmetry and the Egger test (statistical significance threshold: *P* < 0.05) (22). If bias was detected, the trim-and-fill method (22) was used to evaluate its impact on result reliability.

We used Stata 16.0 (Stata Corp, College Station, Texas) for the meta-analysis.

## Results

### Literature search

**Figure 1:**
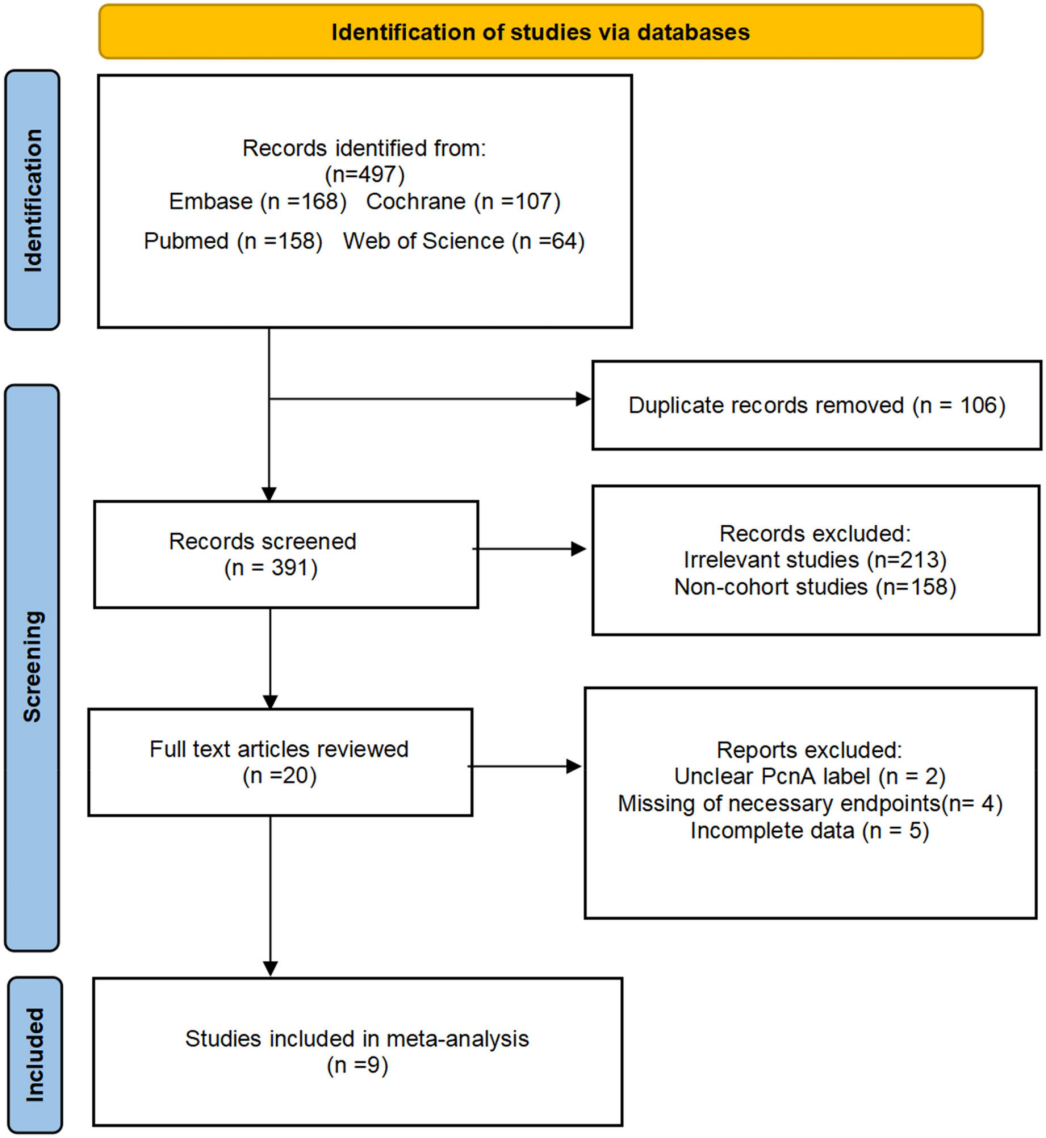
Flow chart. Flow chart of study selection. A total of 497 articles were identified from the PubMed, Embase, Cochrane, and Web of Science databases. After screening, nine articles were included.

A total of 497 articles were identified from the PubMed, Embase, Cochrane and Web of Science databases. After screening, the meta-analysis included 2,431,655 participants. For multiple cardiovascular outcomes in the same study, we analyzed multiple relevant outcomes at the same time if data on overall cardiovascular events were not available. As a result, 13 data points from 9 research reports were included. (Figure 1). Among the included studies, 8were cohort studies and 1were cross-sectional studies, involving a total of 2,383,813 participants. Two studies examined the effects of skipping breakfast on CAD and stroke (23), while one study analyzed its impact on stroke and CDM (15). Overall, five studies evaluated the relationship between skipping breakfast and CAD (14, 23–26), five studies assessed the association with stroke (14, 24, 27–29), and three studies examined CDM (15, 24, 27). Notably, one study focused exclusively on American men (25), another included employee from large companies in Japan (26), a third examined participants in a medical health examination program conducted by Panasonic in Osaka, Japan (24). Additionally, one study involved 980 adult CAD patients who underwent various cardiovascular interventions at a tertiary cardiac hospital in West India between January 2016 and January 2017 (22). The study characteristics are summarized in Table 1. Except for item 1 (Table 3), most of the included studies were of high quality.

**Table 3:** Subgroup analysis of the relationship between of breakfast skipping and CAD, stroke and CDM.

Subgroup analysis of the relationship between of breakfast skipping and CAD, stroke and CDM				
Subgroup	No. of studies	OR (95% CI)	*I*^2^%	p for heterogeneity
CDM	3	1.49 (1.20, 1.84)	0%	0.58
Follow-up time				
≥5 year	2	1.41 (1.12, 1.78)	0%	0.84
<5 year	1	1.87 (1.15, 3.05)	0%	0.58
Total sample size				
<10,000	2	1.77(1.17, 2.67)	0%	0.66
>10,000	1	1.4 (1.10, 1.79)	0%	—
Male				
<50%	2	1.77 (1.17, 2.67)	0%	0.66
Stroke	6	1.12 (1.01, 1.25)	58.16%	0.04
Follow-up time				
≥5 year	4	1.12 (1.03, 1.21)	33.61%	0.21
<5 year	2	1.69 (0.51, 5.6)	85.88%	0.01
Total sample size				
<10,000	1	3.39 (1.4, 8.2)	—	—
>10,000	5	1.1 (1.02, 1.19)	30.71%	0.22
male				<0.01
>50%	2	1.1 (1.05, 1.16)	0.00%	0.82
<50%	4	1.19 (0.92, 1.54)	74.77%	0.01
CAD	5	1.14 (1.05, 1.24)	14.1%	0.32
Follow-up time				
≥5 year	3	1.14(1.04,1.24)	0.00%	0.42
<5 year	2	1.18 (0.93,1.49)	65.74	0.09
Total sample size				
<10,000	2	1.18 (0.93, 1.49)	65.74%	0.09
>10,000	3	1.14 (1.04, 1.24)	0.00%	0.42
Male				
>50%	3	1.19 (1.07, 1.32)	26.11%	0.26
<50%	2	1.05 (0.9, 1.21)	0.00%	0.63

### Risk of cardiovascular diseases

**Figure 2:**
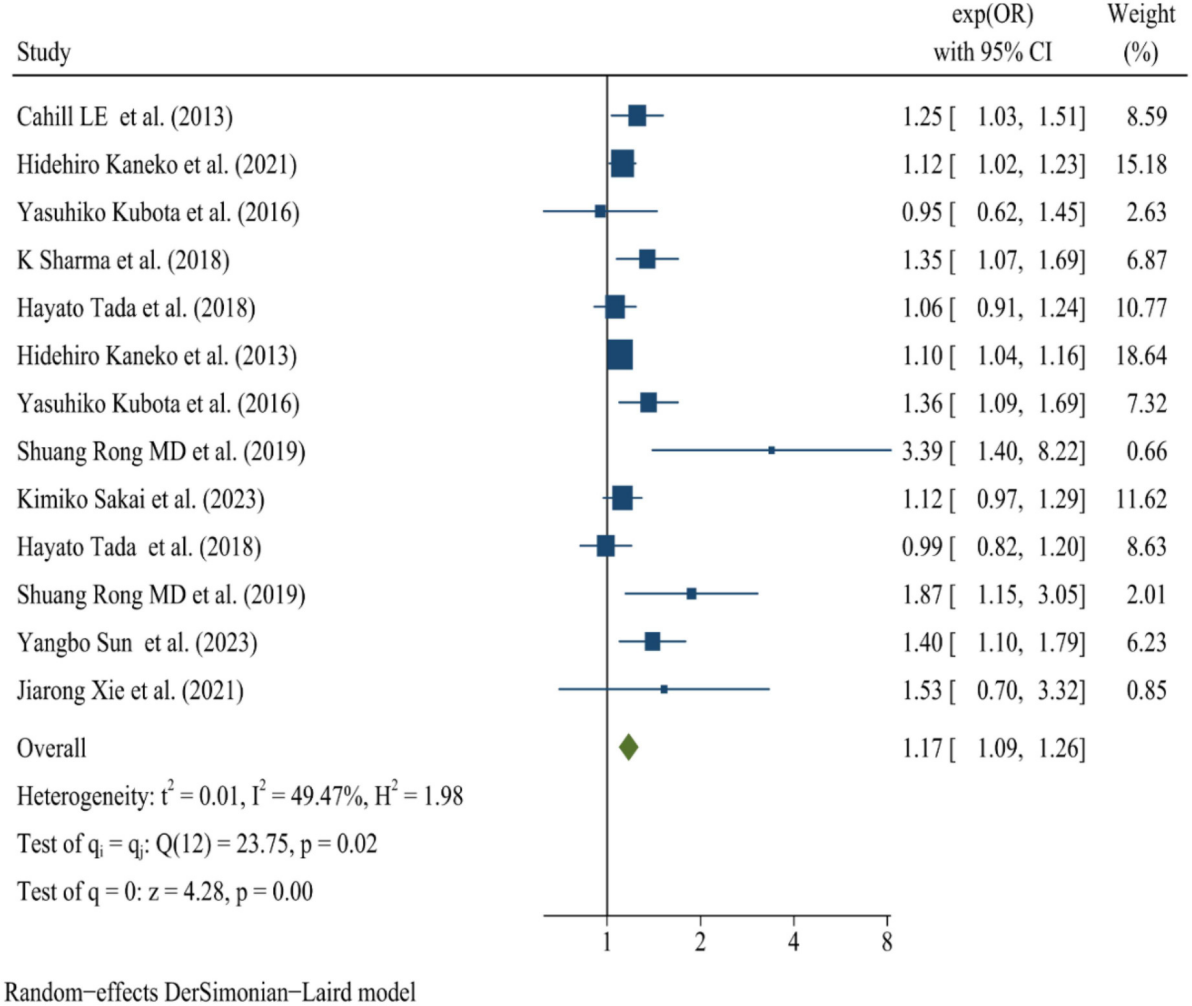
Forest map of skipping breakfast and CVD. Association of skipping breakfast and risk of CVD. OR of studies are denoted by Blue square. The lines of individual studies represent the 95% CI. The Green diamond shape represents the 95% CI of pooled OR. A random effects model was used for the meta-analysis. When a study reports multiple cardiovascular outcomes but lacks data on overall cardiovascular events, we analyzed relevant outcomes concurrently. For instance, in the cohort study by Hidehiro Kaneko et al., both coronary artery disease (CAD) and stroke were assessed separately, resulting in their repeated inclusion in the Forest map.

Skipping breakfast is significantly related to the increased risk of CVD (Figure 2). A summary analysis of 13 comparisons (18, 19, 24–30) reveals that skipping breakfast is significantly associated with an increased risk of CVD, with an OR of 1.17 (95% CI: 1.09–1.26). Evidence indicates moderate heterogeneity (*I*^2^ = 49.47%). Stratified analyses by outcome type show that skipping breakfast is significantly associated with CAD (OR: 1.14, 95% CI: 1.05–1.24), CDM (OR: 1.49, 95% CI: 1.20–1.84), and stroke (OR: 1.15, 95% CI: 1.01–1.30).

### Sensitivity analysis

**Figure 3:**
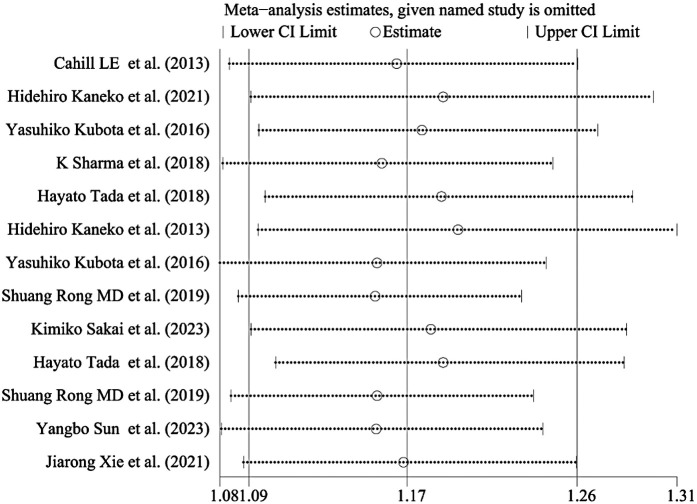
Sensitivity analysis. To assess the relative influence of individual studies on the pooled estimate, sensitivity analyses were performed by systematically omitting one study at a time. When a study reports multiple cardiovascular outcomes but lacks data on overall cardiovascular events, we analyzed relevant outcomes concurrently. For instance, in the cohort study by Hidehiro Kaneko et al., both coronary artery disease (CAD) and stroke were assessed separately, resulting in their repeated inclusion in the sensitivity analysis.

Sensitivity analysis showed that when any study was excluded, there was no opposite outcome in the combined results, indicating the stability of the outcome (Figure 3).

### Publication bias

**Figure 4:**
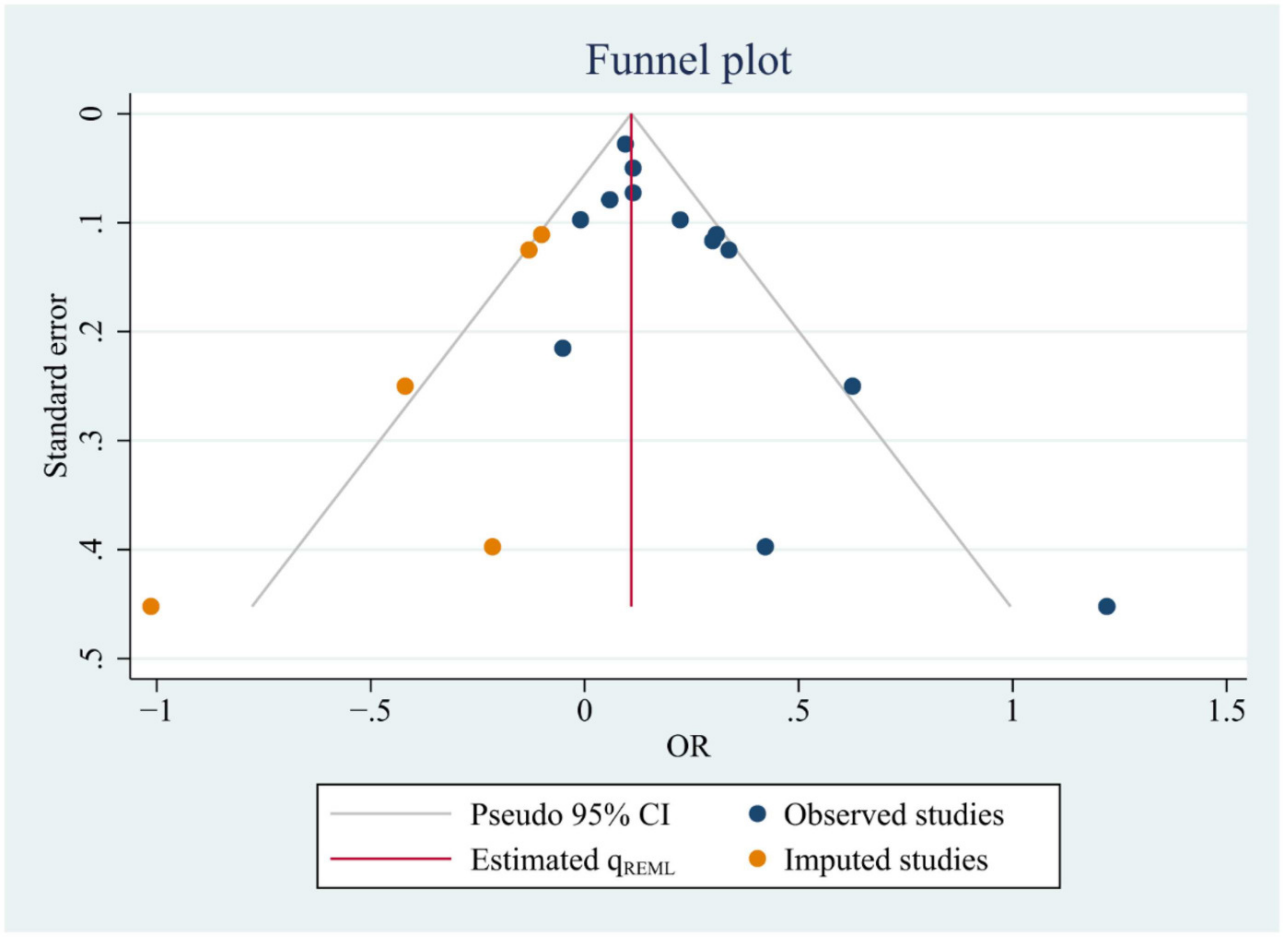
Funnel plot. The funnel diagram, following the application of bias-cutting techniques, is used to assess the potential for publication bias.

Publication bias was assessed across the 13 comparisons using the Egger test (*p* = 0.02), which indicated significant bias. To address this, we applied the trimming and filling method (Figure 4), a funnel chart-based approach. This analysis suggested that, in theory, five missing studies should be included. After applying the trimming and filling procedure, the funnel chart became more symmetrical. Importantly, the trimming and filling analysis did not alter the direction of the results, indicating that the findings are reliable.

### Subgroup analysis

**Figure 5:**
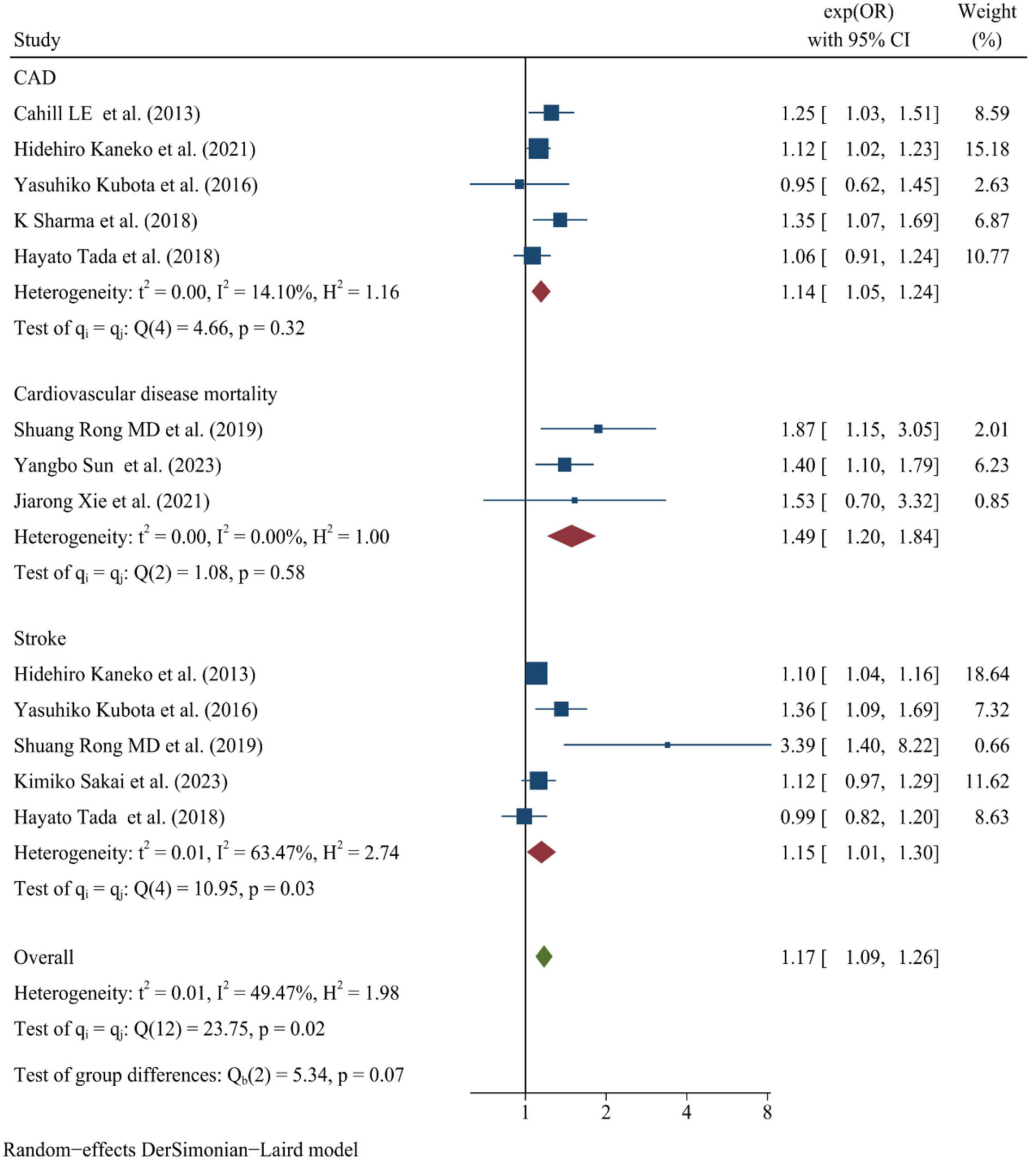
Subgroup analysis of disease categories. We estimated the combined OR by stratifying all estimates according to disease type. The horizontal lines for individual studies represent the 95% CI. The green diamond denotes the 95% CI for the pooled OR, while the red diamond represents the summary estimate from the stratification analysis of different diseases. A random-effects model was used for the meta-analysis. When a study reported multiple cardiovascular outcomes without data on overall cardiovascular events, we analyzed the relevant outcomes concurrently. For example, in the cohort study by Hidehiro Kaneko et al., CAD and stroke were assessed separately, leading to their repeated inclusion in the subgroup analysis.

**Figure 6:**
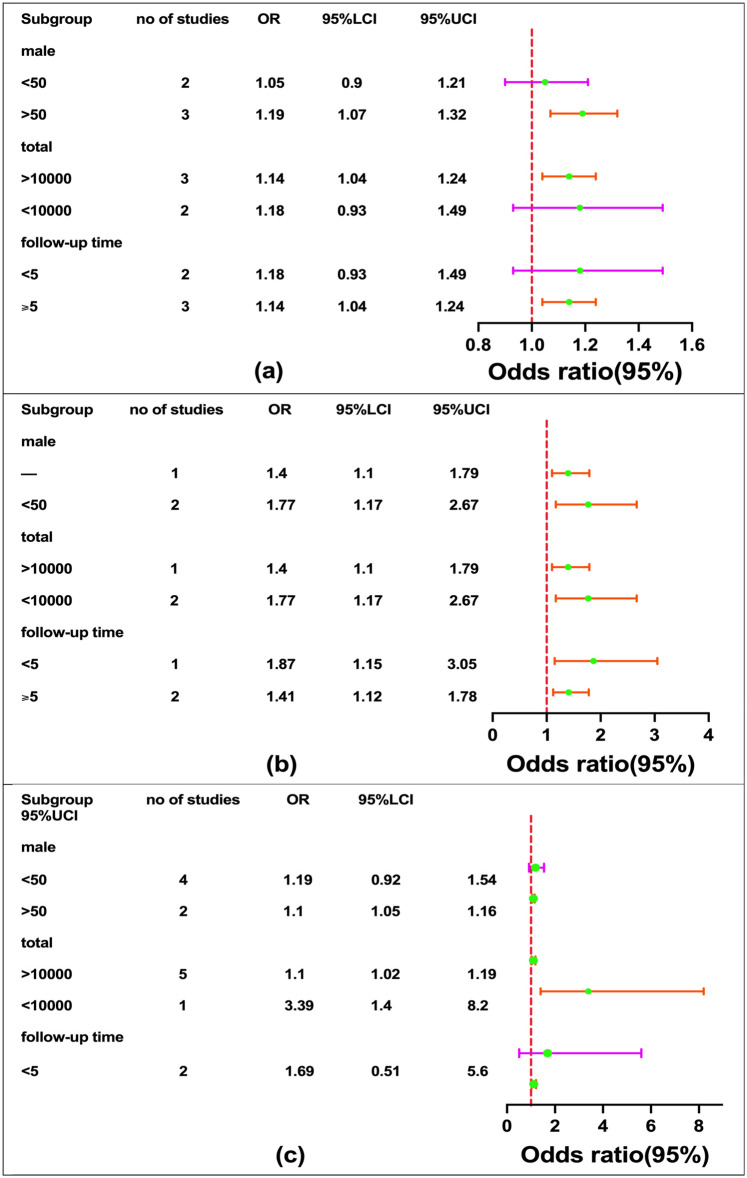
Subgroup analysis. Among these, a, b, and c represent subgroup analyses of CAD, CDM, stroke adjusted for follow-up duration, total sample size, and proportion of male participants.

In the additional meta-analysis, we estimated the combined OR by stratifying all estimates according to disease type (Figure 5). In all subgroup analyses using multivariate adjustment models (Figure 6), the detailed summary is provided in Table 3.

Subgroup analysis indicated that follow-up durations exceeding five years or sample sizes larger than 10,000 produced more precise results, evidenced by narrower confidence intervals, greater stability, and reduced heterogeneity. This identifies follow-up duration and sample size as potential sources of heterogeneity. When analyzing studies with follow-up periods under five years, the association between skipping breakfast and the incidence of stroke or CAD weakened, yielding an odds ratio (OR) of 1.69 (95% CI: 0.51, 5.60) for stroke and 1.18 (95% CI: 0.93, 1.49) for CAD. A similarly weakened correlation for CAD was observed in studies with fewer than 10,000 participants, with an identical OR of 1.18 (95% CI: 0.93, 1.49). Consequently, in studies with either a follow-up duration of less than five years or a sample size below 10,000, the relationship between skipping breakfast and CAD remains consistent and does not contribute to result heterogeneity.

Additionally, when the proportion of male participants is less than 50%, the association between skipping breakfast and the incidence of stroke or CAD weakened The OR for skipping breakfast and stroke is 1.19 (95% CI: 0.92, 1.54), and the OR for skipping breakfast and CAD is 1.05 (95% CI: 0.90, 1.21), which warrants further discussion.

## Discussion

We conducted a meta-analysis using CVD risk estimates from nine studies. The results revealed a significant association between skipping breakfast and increased CVD incidence, with a summary OR of 1.17 (95% CI: 1.09–1.26). Subgroup analysis by disease type showed that the association between skipping breakfast and CAD, stroke, and CDM remained significant. Notably, when the analysis was stratified by adjustment for different covariates, the association between skipping breakfast and stroke or CAD strengtheneded over time. This suggests that the impact of skipping breakfast on cardiovascular health may be a long-term process. Furthermore, when the total sample size was fewer than 10,000 participants, the association between skipping breakfast and CAD diminished, suggesting that the results might be affected by small sample bias.

The current meta-analysis aligns with the findings of this study, indicating that skipping breakfast is associated with an elevated risk of CVD, stroke, CAD, and CDM. Takagi et al. noted that the definition of skipping breakfast varied by frequency, and they thoroughly examined the effects of different frequencies on CVD (31). Bonnet et al. conducted a meta-analysis of randomized controlled trials on breakfast skipping, providing limited data on its impact on cardiometabolic parameters (32). However, their study's short 4-week duration and lack of measurement in most analytical tests reduce its persuasiveness compared to the cohort study presented here (33). Zhi-hui et al. broadly defined skipping breakfast as eating breakfast ≤3 times/week and found it significantly lowers the risk of CVD and metabolic diseases, including type 2 diabetes, obesity, and metabolic syndrome. However, their analysis included only five cohort studies examining the association between breakfast skipping and coronary heart disease, stroke, and cardiovascular mortality. The limited data weakens its reliability compared to this study. This study classifies CVD into three subtypes—CAD, stroke, and CDM—each supported by at least three studies, allowing for a more robust subgroup analysis of breakfast skipping's cardiovascular effects. Unlike previous meta-analyses, this research incorporates a larger cohort dataset than Zhi-hui et al. and avoids the methodological constraints of Bonnet et al.'s short-term randomized trials.

Gender differences significantly influence the impact of skipping breakfast on the risk of stroke and CHD, with men at higher risk than women. These differences may primarily stem from lifestyle habits, physiological mechanisms, and genetic factors. Regarding lifestyle, cardiovascular risk factors such as smoking, and alcohol consumption are more prevalent in men (30). From a physiological standpoint, hormonal differences play a critical role. Estrogen provides a protective effect on the blood vessels of premenopausal women by regulating potassium channels activated by nitric oxide (NO) and calcium ions, thereby reducing the risk of arrhythmias and CVD (34–37). Additionally, women's higher carotid artery outflow/inflow ratio reduces the risk of energy loss and local vascular stress (38). In terms of fibrosis, men typically have higher levels of fibrotic markers (39, 40), which promote the progression of cardiovascular diseases. Regarding cellular mechanisms, the mortality rate of cardiac muscle cells in aging men is higher than in women, leading to more severe heart failure in men (41). Fat distribution differences also contribute significantly, as men tend to accumulate more visceral fat, which is strongly associated with insulin resistance and cardiovascular events (42, 43). Finally, genetic factors, including specific mutations on the Y chromosome, are linked to hypertension and may help explain the higher incidence of CVD in men (44).

The relationship between skipping breakfast and the risk of CVD primarily manifests through several mechanisms. Skipping breakfast impairs physiological insulin secretion, leading to disruptions in glucose homeostasis, which can contribute to the onset of diabetes. Diabetes often results in abnormal blood lipid profiles, including elevated triglycerides and reduced HDL-C. Triglycerides, primarily indirectly measured, are broken down into cholesterol residues, which are further converted into Apo-B48-containing lipoproteins. When these particles enter the arterial wall, they induce low-grade inflammation, foam cell formation, and the development of atherosclerotic plaques, ultimately increasing the risk of CVD and CDM (45). Moreover, elevated cholesterol levels directly contribute to the deposition of cholesterol in blood vessel walls, impeding blood flow and oxygen delivery (46), which exacerbates CVD and promotes the progression of atherosclerosis (47). Habitual breakfast skipping is also associated with elevated systemic inflammatory markers, such as C-reactive protein and glycoprotein acetyl, and these inflammatory mediators are positively correlated with blood pressure (48). Hypertensive patients exhibit increased levels of interleukin-6 (IL-6), C-reactive protein, and other inflammatory markers (6–9). Chronic fluctuations in blood pressure can damage the endothelium and elastic fibers, ultimately increasing arterial stiffness, causing target organ damage, and elevating the risk of CDM and CVD (48).

Following this meta-analysis, individuals who skip breakfast should be more vigilant in assessing their CVD risk. Targeted investigations into specific types of CVD are also recommended, with the goal of incorporating more risk factors into clinical evaluations.

## Advantages and limitations

In this meta-analysis, we categorize CVD into three subtypes: CAD, stroke, and CDM, each of which is supported by three or more research reports. Furthermore, we conduct subgroup analyses for each subtype, adjusting for various covariates to better elucidate the relationship between skipping breakfast and CVD. We employ multiple statistical methods, including OR, 95% CI, publication bias assessment, and sensitivity analysis, to enhance the credibility and reliability of the results. The limitations of our meta-analysis should be acknowledged. First, data on skipping breakfast were obtained from self-reported questionnaires or interviews in all studies, making it susceptible to reporting inaccuracies and measurement errors. Additionally, breakfast habits may change over the follow-up period, further contributing to misclassification. Second, few studies provided a precise definition of breakfast, and global definitions of breakfast remain inconsistent (49). The lack of standardized definitions may introduce bias and heterogeneity in meta-analyses. While subgroup analyses comparing complete skippers (0 times/week) and occasional skippers (1–4 times/week) can help, existing data remain insufficient to resolve definition-related heterogeneity. Establishing a uniform breakfast definition would improve investigations of its association with health outcomes. Third, this meta-analysis focuses solely on the impact of skipping breakfast, without accounting for the specific foods and beverages consumed during this meal. Different breakfast items, such as cereals, red meat, eggs, milk, and coffee, may have varying effects. Consequently, we are unable to offer recommendations on the optimal composition or quantity of breakfast. Fourth, while most studies adjusted for major covariates, unmeasured confounding factors may still influence the findings. Fifth, all included studies were conducted in Western developed countries, limiting the generalizability of the results to populations in other regions. Given that dietary patterns vary significantly across countries (50), regional differences should be considered in future research. Sixth, this study is limited by substantial heterogeneity. Subgroup analyses failed to identify significant sources of variation, though potential factors may include population characteristics, study designs, and confounding variables. Current data limitations prevent further investigation of these aspects. Future prospective cohort studies are needed to better understand the sources of heterogeneity. Finally, as our findings are primarily based on observational studies, it is not possible to establish a causal relationship.

## Conclusion

This meta-analysis demonstrates that skipping breakfast significantly increases the risk of CVD and CDM. Regular breakfast consumption is recommended to support cardiovascular health.

## Data Availability

The original contributions presented in the study are included in the article/Supplementary Material, further inquiries can be directed to the corresponding author.
